# Peripheral Electrical Stimulation Paired With Movement-Related Cortical Potentials Improves Isometric Muscle Strength and Voluntary Activation Following Stroke

**DOI:** 10.3389/fnhum.2020.00156

**Published:** 2020-05-15

**Authors:** Sharon Olsen, Nada Signal, Imran K. Niazi, Usman Rashid, Gemma Alder, Grant Mawston, Rasmus B. Nedergaard, Mads Jochumsen, Denise Taylor

**Affiliations:** ^1^Health and Rehabilitation Research Institute, Auckland University of Technology, Auckland, New Zealand; ^2^Centre for Chiropractic Research, New Zealand College of Chiropractic, Auckland, New Zealand; ^3^Center for Sensory-Motor Interaction, Department of Health Science and Technology, Aalborg University, Aalborg, Denmark; ^4^Department of Clinical Medicine, Aalborg University, Aalborg, Denmark; ^5^Mech-Sense, Department of Gastroenterology and Hepatology, Aalborg University Hospital, Aalborg, Denmark

**Keywords:** paired associative stimulation, movement related cortical potential, neuromodulation, stroke, muscle strength, voluntary activation, twitch interpolation

## Abstract

**Background:**

Endogenous paired associative stimulation (ePAS) is a neuromodulatory intervention that has potential to aid stroke recovery. ePAS involves pairing endogenous electroencephalography (EEG) signals known as movement-related cortical potentials (MRCPs), with peripheral electrical stimulation. Previous studies have used transcranial magnetic stimulation (TMS) to demonstrate changes in corticomotor excitability following ePAS. However, the use of TMS as a measure in stroke research is limited by safety precautions, intolerance, and difficulty generating a measurable response in more severely affected individuals. We were interested in evaluating the effect of ePAS using more feasible measures in people with stroke. This study asks whether ePAS produces immediate improvements in the primary outcomes of maximal voluntary isometric contraction (MVIC) and total neuromuscular fatigue of the dorsiflexor muscles, and in the secondary outcomes of muscle power, voluntary activation (VA), central fatigue, peripheral fatigue, and electromyography activity.

**Method:**

In this repeated-measures cross-over study, 15 participants with chronic stroke completed two interventions, ePAS and sham, in a randomized order. During ePAS, 50 repetitions of visually cued dorsiflexion were completed, while single pulses of electrical stimulation were delivered to the deep branch of the common peroneal nerve. Each somatosensory volley was timed to arrive in the primary motor cortex at the peak negativity of the MRCP. Univariate and multivariate linear mixed models were used to analyze the primary and secondary data, respectively.

**Results:**

There was a statistically significant increase in dorsiflexor MVIC immediately following the ePAS intervention (mean increase 7 N), compared to the sham intervention (mean change 0 N) (univariate between-condition analysis *p* = 0.047). The multivariate analysis revealed a statistically significant effect of ePAS on VA of the tibialis anterior muscle, such that ePAS increased VA by 7 percentage units (95% confidence interval 1.3–12.7%). There was no statistically significant effect on total neuromuscular fatigue, muscle power, or other secondary measures.

**Conclusion:**

A single session of ePAS can significantly increase isometric muscle strength and VA in people with chronic stroke. The findings confirm that ePAS has a central neuromodulatory mechanism and support further exploration of its potential as an adjunct to stroke rehabilitation. In addition, the findings offer alternative, feasible outcome measures for future research.

**Clinical trial registration:**

Australia New Zealand Clinical Trials Registry ACTRN12617000838314 (www.anzctr.org.au), Universal Trial Number U111111953714.

## Introduction

Endogenous paired associative stimulation (ePAS) is an innovative neuromodulatory intervention that has potential to improve recovery following stroke (Mrachacz-Kersting et al., 2016, 2019). Based on traditionally delivered paired associative stimulation (PAS) (Carson and Kennedy, 2013; Suppa et al., 2017; Alder et al., 2019), ePAS involves the pairing of two components: (i) movement-related cortical potentials (MRCPs), which are endogenous electroencephalography (EEG) signals produced during imagined or voluntary movement, and (ii) peripheral electrical stimulation (Mrachacz-Kersting et al., 2012). The intervention is carefully timed so that the afferent signals created by the peripheral electrical stimulation arrive in the primary motor cortex (M1) when it is being naturally activated by imagined or executed movement; this pairing of afferent and efferent signals is thought to induce associative plasticity (Mrachacz-Kersting et al., 2012, 2016). Previous research, which measured pre- to post-intervention changes in corticomotor excitability using transcranial magnetic stimulation (TMS), has shown that ePAS produces an immediate increase in corticomotor excitability (Mrachacz-Kersting et al., 2012, 2016, 2019; Niazi et al., 2012; Olsen et al., 2017; Mrachacz-Kersting and Aliakbaryhosseinabadi, 2018). Two studies have demonstrated this excitatory effect in people with stroke who have a measurable motor evoked potential (MEP) in a resting muscle (Mrachacz-Kersting et al., 2016, 2019). However, the use of TMS as an outcome measure in the wider stroke population is limited by safety precautions, poor tolerance to stimulation, and the inability to generate a MEP in certain individuals, particularly those with greater motor impairment (Heald et al., 1993; Rossi et al., 2009). Therefore, we were interested in exploring the immediate effects of ePAS using more feasible outcome measures, to ensure that future research could be generalized to the broader stroke population.

A range of measures of motor impairment and neurophysiology have potential to demonstrate the neuromodulatory effects of ePAS. Previous studies have illustrated changes in isometric muscle strength (Tanaka et al., 2009, 2011; Sohn et al., 2013; Hazime et al., 2017), neuromuscular fatigue (Benwell et al., 2006; Cogiamanian et al., 2007; Abdelmoula et al., 2016; Lattari et al., 2016), and muscle power (Lattari et al., 2017), in response to other neuromodulatory interventions such as repetitive TMS (rTMS) and transcranial direct current stimulation (tDCS). For measuring changes in neurophysiology, voluntary activation (VA), which uses twitch interpolation to measure the central nervous system drive to a muscle during a voluntary contraction (Gandevia et al., 1996), is decreased in the hemiparetic limb following stroke (Traversa et al., 2000; Newham and Hsiao, 2001; Horstman et al., 2008; Stinear et al., 2015) and has been shown to increase following 4 days of tDCS in healthy participants (Frazer et al., 2016). Additional neurophysiological measures include EMG measures, such as EMG amplitude, which has been shown to increase in healthy participants following tDCS to the M1 (Krishnan et al., 2014). These impairment-based and neurophysiological measures offer potential outcomes to explore the effects of ePAS.

The objective of the following study was to establish the immediate effects of ePAS on the primary outcomes of isometric muscle strength and total neuromuscular fatigue of the ankle dorsiflexor muscles, in comparison to an attention- and dose-matched sham intervention, in people with chronic stroke. These primary outcome measures were chosen because they had the most literature to support their potential to illustrate the effects of neuromodulation (Benwell et al., 2006; Tanaka et al., 2011; Sohn et al., 2013). The study also investigated, but was not powered to detect, the effects of ePAS on a range of secondary measures of impairment (simple fatigue index, muscle power) and neurophysiology (VA, central fatigue, peripheral fatigue, EMG activity, and corticomotor excitability). Measures of corticomotor excitability using TMS were included to confirm previous findings but were only collected if deemed safe and tolerable for participants, to ensure this had no influence on study recruitment.

## Materials and Methods

### Study Design

**FIGURE 1:**
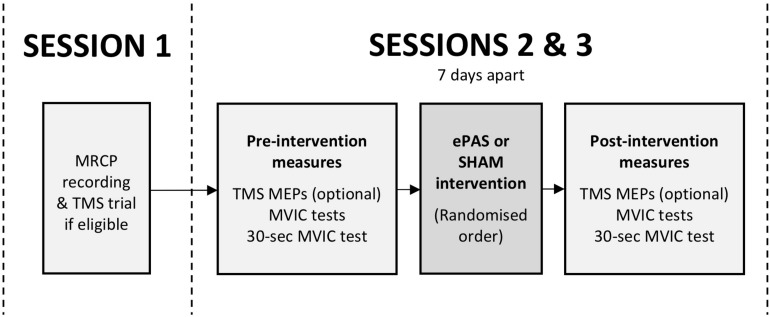
Study flow, including preparatory session (session 1) and intervention sessions (sessions 2 and 3). Outcome measures included TMS motor evoked potentials (MEPs), maximal voluntary isometric contractions (MVICs), and an MVIC sustained for 30 s.

This study was a double-blind, within-subject, repeated-measures, cross-over experiment. Participants attended three sessions (see Figure 1). The first session was a preparation session, during which the MRCP was recorded, and TMS was trialed if it had been determined to be safe during screening (see section “Participants”). The second and third sessions were intervention sessions, separated by 7 days, during which participants received one ePAS intervention and one sham intervention, in a randomized order. The randomization order was based on a *true* random integer set generated from random.org. Outcome measures were collected immediately before and immediately after each intervention.

### Participants

Volunteers were considered for inclusion if they were over 18 years of age, had had a single stroke more than 6 months previously, and had a hemiparesis affecting ankle dorsiflexion movement. Participants were excluded if they had significant cognitive, perceptual, or communication deficits, cerebellar stroke, contra-indications to peripheral electrical stimulation, an inability to generate ankle dorsiflexor force in the ankle dynamometer, or medical conditions that would impact the safety of the participant or their ability to complete the protocol. TMS-induced measures of corticomotor excitability were an optional outcome measure in this study, and therefore participants were screened for the following contraindications and precautions: epilepsy or seizures, pacemaker, metal implants above the chest level, recurring headaches, skull fracture or defects, head injury within the last 6 months, and medications that significantly lower seizure threshold. A sample size calculation based on available evidence (Benwell et al., 2006; Tanaka et al., 2011; Sohn et al., 2013) estimated a sample size of ten participants would enable the detection of statistically significant differences in within-session measures of isometric muscle strength and total neuromuscular fatigue (80% power, alpha 0.05). This sample size was increased to 15 due to limitations in the available evidence and risk of dropouts. Participants were asked to refrain from consuming caffeine or exercising prior to the testing sessions. All participants provided written informed consent and the study received ethical approval from the Health and Disability Ethics Committees (17/NTB/80). The study was registered with the Australian New Zealand Trials Registry (Trial ID ACTRN12617000838314).

### Randomization and Blinding

Participants were blinded to randomization and were unaware of the use of a sham intervention. This was achieved by advising participants that two different interventions would be delivered in sessions 2 and 3, and that one would be moderate-intensity, and the other low-intensity, but the participant would not be aware of which intervention was being delivered. The researchers performing outcome assessments and data processing remained blinded to randomization.

### MRCP Recording and TMS Trial (Session 1)

Participants sat in a height-adjustable chair with their hemiparetic leg supported with the knee in approximately 50° of flexion. An EEG cap (40 channel Quik-cap, Ag/AgCl electrodes, Compumedics Neuroscan) was mounted with the Cz electrode placed midway between the nasion and the inion in the sagittal plane, and midway between each tragus in the coronal plane. A sterile blunt needle was used to abrade skin and apply conductive gel to the FP1, F3, Fz, F4, C3, Cz, C4, P3, Pz, P4, and ground electrodes (impedance < 5 kΩ). A reference electrode (Blue sensor N, Ambu, Denmark) was placed on the right mastoid process. Participants completed two sets of 25 repetitions of voluntary ankle dorsiflexion while following a visual cue displayed on a computer monitor which prompted them to (1) bring their attention to the screen [2–3 seconds(s)], (2) prepare for movement (3 s), (3) dorsiflex their ankle (1.5 s), and then (4) rest (6–8 s). The ankle movement was ballistic, and against gravity but not resistance. An amplifier recorded continuous EEG data with a sampling frequency of 500 Hz and 32 bits accuracy (NuAmps 40 channel digital EEG and ERP amplifier and SCAN 4.5 software, Compumedics Neuroscan, United States).

**FIGURE 2:**
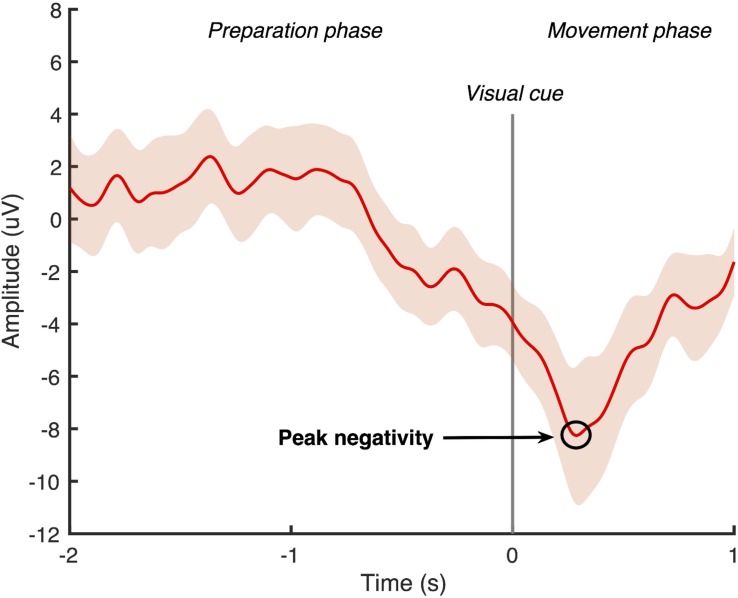
Epoch-averaged MRCP with 95% confidence interval for one participant with chronic stroke (epochs = 47). Visual cue to move is delivered at 0 s and peak negativity occurred 286 ms after visual cue.

Using Matlab software (Mathworks, United States), the continuous EEG data was band-pass filtered with a zero-phase shift infinite impulse response Butterworth filter at 0.05–5 Hz. A large Laplacian filter was applied to all channels except the FP1 and ground to create a surrogate channel centered around the Cz electrode (Jochumsen et al., 2015a). The filtered signal was then divided into 50 epochs, which were time-locked to the cue to dorsiflex the ankle. Each epoch contained a 2-second “movement preparation phase” that ended with the cue to dorsiflex the ankle, followed by a 1-second “movement phase.” Two researchers experienced in viewing MRCP data independently screened all epochs and manually rejected any that contained eye-movement artifacts or were not the characteristic MRCP shape (Mrachacz-Kersting et al., 2012, 2016, 2019; Olsen et al., 2017). An average of the remaining MRCPs was computed from the surrogate channel, and the most negative point of the waveform was identified as the peak negativity (see example Figure 2). If the peak negativity timing differed by more than 10 ms between the two researchers, they repeated the MRCP processing together. If the peak negativity timing differed by less than 10 ms, an average of the two measures was used. The timing of the peak negativity of the average MRCP was calculated with respect to the onset of the cue to dorsiflex the ankle and this timing was used in the delivery of the subsequent ePAS interventions in sessions 2 and 3.

Following the MRCP recording, those participants who were eligible were given the opportunity to trial receiving TMS, to determine their ability to tolerate the stimulation and the presence of a MEP in the tibialis anterior muscle (TA) in response to stimulation. If a MEP was not measurable, or if TMS was not deemed safe, or not deemed tolerable in terms of comfort or the extra time required, sessions 2 and 3 were conducted without TMS measures.

### Outcome Measures (Sessions 2 and 3)

Outcome measures were recorded immediately pre- and post-intervention. Following TMS measures, the other outcomes were collected during a maximum voluntary isometric contraction (MVIC) task and then a 30-s MVIC task. There were two primary outcomes: MVIC, and total neuromuscular fatigue measured with the area under the curve (AUC) fatigue index. The primary and secondary outcomes are listed in Table 1.

**TABLE 1:** Outcome measures.

**Task**	**Outcome measures**	**Primary or secondary**	**Equations (if applicable)**
At rest	TA motor evoked potentials (MEPs)	Secondary	
MVIC task	MVIC dorsiflexors [Newtons (N)]	Primary	
	Time to 90% MVIC dorsiflexors [seconds (s)]	Secondary	
	Rate of force development (ROFD) 0–200 ms dorsiflexors (N/s)	Secondary	
	TA EMG amplitude [Volts (V)]	Secondary	
30-s MVIC task	AUC fatigue index (%)	Primary	A. (1-AUC⁢from⁢ 5⁢sec⁢to⁢end⁢taskMaximum⁢force×length⁢of⁢task)× 100
	Simple fatigue index (%)	Secondary	B. (1-Mean⁢force⁢final⁢ 3⁢epochsMean⁢force⁢initial⁢ 3⁢epochs)× 100
	Decline in EMG amplitude (%)	Secondary	$C.\;\left(1-\frac{\mathrm{RMS}\,\mathrm{final}\,\mathrm{epoch}}{\mathrm{RMS}\,\mathrm{initial}\,\mathrm{epoch}}\right)\times 100$
	Decline in EMG median frequency (MF) (%)	Secondary	$D.\;\left(1-\frac{\mathrm{MF}\,\mathrm{final}\,\mathrm{epoch}}{\mathrm{MF}\,\mathrm{initial}\,\mathrm{epoch}}\right)\times 100$
	Voluntary activation (VA) (%)	Secondary	$E.\;\left(1-\frac{\mathrm{Initial}\,\mathrm{active}\,\mathrm{twitch}}{\mathrm{Initial}\,\mathrm{resting}\,\mathrm{twitch}}\right)\times 100$
	Central fatigue (%)	Secondary	$F.\;\mathrm{VA}\mathrm{end}\mathrm{task}=\left(1-\frac{\mathrm{Final}\,\mathrm{active}\,\mathrm{twitch}}{\mathrm{Final}\,\mathrm{resting}\,\mathrm{twitch}}\right)\times 100\left[\mathrm{cpsbreak}\right]G.\;\mathrm{Central}\mathrm{fatigue}=\mathrm{VA}-\mathrm{VA}\mathrm{end}\mathrm{task}$
	Peripheral fatigue (%)	Secondary	$H.\;\left(1-\frac{\mathrm{Final}\,\mathrm{resting}\,\mathrm{twitch}}{\mathrm{Initial}\,\mathrm{resting}\,\mathrm{twitch}}\right)\times 100$

### Procedures (Sessions 2 and 3)

#### TMS

For all procedures, participants sat in an orthopedic height-adjustable armchair. For TMS measurements, the ankle was supported on foam padding and rested in slight plantarflexion. Following skin preparation, EMG surface electrodes (Blue sensor N, Ambu, Denmark) were placed on the skin surface over the hemiparetic TA. The two detection electrodes were placed 2 cm apart, a third of the way along the line between the head of the fibula and the tip of the medial malleolus^1^, and a reference electrode was placed on the lower third of the anterior border of the tibia. Impedance was checked with a digital multimeter with the aim of ensuring a level below 30 kΩ (Konrad, 2005). If the impedance was higher, additional abrasion was applied, but only if the researcher deemed skin integrity would not be compromised. Single monophasic pulses of TMS (Magstim 200, Magstim Co., Dyfed, United Kingdom) were applied with a double cone coil to determine the TA hotspot and resting motor threshold (RMT) according to a previously described method (Olsen et al., 2017). Single monophasic pulses were applied at 120% RMT, to produce ten TA MEPs. MEPs were recorded with TA EMG, then amplified (×500) (AMT-8, Bortec Biomedical, Canada) and sampled at 2000 Hz using a data acquisition board (Micro 1401, CED, United Kingdom) and Signal software (CED, United Kingdom).

#### MVIC Task

**FIGURE 3:**
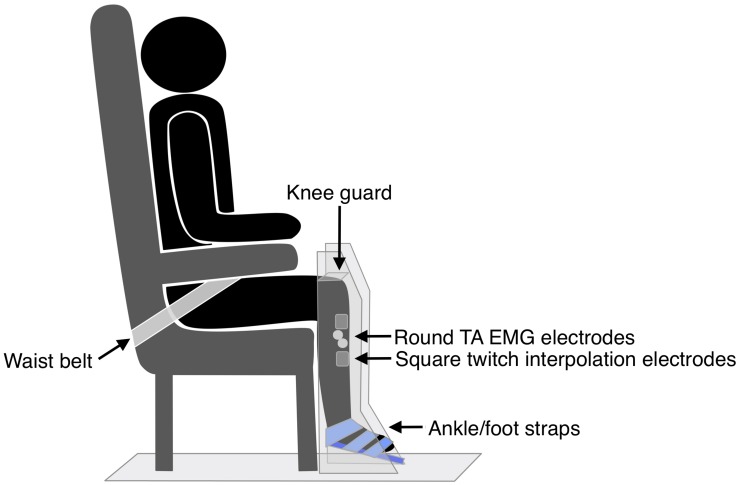
Set up for strength and fatigue outcome measures.

The hemiparetic leg was positioned on a purpose-built dynamometer that was secured to the floor, with a fixed footplate angled 25° to the floor (see Figure 3). The dynamometer measured isometric ankle dorsiflexion/plantarflexion force via a load cell (capacity 100 lbs, error < 0.05%, Model MLP100, Transducer Techniques, CA, United States). The hemiparetic leg was secured with three straps over the ankle, metatarsals, and toes (Siddiqi et al., 2015), and a knee guard and waist belt were secured to prevent hip flexion and forward motion of the pelvis. TA EMG electrodes were positioned as for TMS measures. Participants completed two submaximal practices of voluntary isometric dorsiflexion followed by a 2-min rest. Participants then performed three 4–5 s MVICs, with 2-min rests in between. Participants were instructed to “pull as fast and hard as possible” and received loud verbal encouragement throughout the task and real-time visual feedback of their force production on a computer monitor.

#### 30-s MVIC Task

**FIGURE 4:**
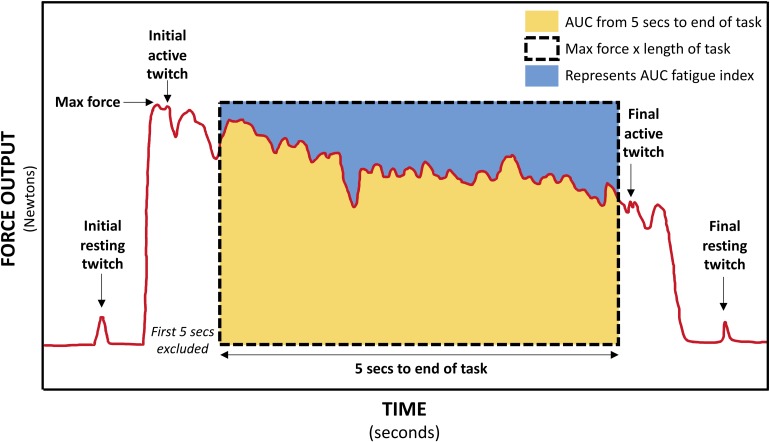
Schematic of force trace during 30-s MVIC showing muscle twitches applied for calculation of voluntary activation (VA), central fatigue, and peripheral fatigue, and the areas used to calculate the area under curve (AUC) fatigue index (represented by the blue area above the force–time curve).

Two muscle stimulation electrodes (5 × 5 cm PALS, Axelgaard, United States) were placed over the hemiparetic TA muscle, just below the tibial plateau, and approximately midway down the tibia. These electrodes were used for applying muscle twitches during the 30-s MVIC; the twitch forces produced were then utilized in the calculation of VA, central fatigue, and peripheral fatigue measures. The optimum position of the electrodes was determined by applying single 1 ms pulses of electrical stimulation (DS7A, Digitimer Ltd., United Kingdom) to the TA muscle at increasing intensity until a twitch contraction was palpable at the tendon of the TA muscle, without concurrent twitches in the tendons of the peroneal, plantar flexor, or toe extensor muscles. The location of the electrodes was marked on the skin with an indelible pen. The intensity of supramaximal muscle stimulation was established by applying doublet 1 ms pulses (10 ms inter-pulse interval, 300 V) to the resting TA muscle in increasing 5 mA increments until there was a plateau in force production (Todd et al., 2004). The amplitude reached was identified as 100% intensity, and subsequent muscle stimulation was delivered at 120% of this value. Following a 5-min rest period, participants completed a single 30-s MVIC while receiving loud continuous verbal encouragement and watching a real-time display of their force. Using manual triggering, doublet 1 ms pulses (10 ms inter-pulse interval, 300 V) were applied to the TA during the initial resting period (initial resting twitch), at the start of the fatigue task once a plateau in force had been reached (initial active twitch), at the end of the fatigue task (final active twitch), and after task completion (final resting twitch) (Figure 4). As various methods are used in the literature (Todd et al., 2004; Klass et al., 2005; Baudry et al., 2007; Klein et al., 2013; Huang et al., 2015), this pulse method had been determined during a piloting period where a comparison was made between a doublet pulse, 5-pulse burst (50 Hz) and 10-pulse burst (50 Hz) to determine the most effective but comfortable method. Two participants could not tolerate higher intensities of stimulation and so the maximum tolerated intensity was used (Huang et al., 2015).

Force and TA EMG data were collected simultaneously during the three MVICs and the 30-s MVIC task. Force signals were amplified (with an adjustable gain of 200, 500, or 1000 depending on amplitude) (Forza, OT Bioelettronica, Italy) and sampled at 1961 Hz using a data acquisition board (Micro 1401, CED, United Kingdom) and Spike2 software (CED, United Kingdom). TA EMG data was amplified (×500) (AMT-8, Bortec Biomedical, Canada), then sampled at 1961 Hz using a data acquisition board (Micro 1401, CED, United Kingdom) and Spike2 software (CED, United Kingdom).

#### Interventions

The participant’s foot was supported on foam padding which allowed for free ankle movement (hip flexion ≈70°, knee flexion ≈50°). A movable bar electrode with electrode gel was placed over the deep branch of the common peroneal nerve (dCPN), 2–5 cm anterior and inferior to the head of the right fibula. For most participants this required that the proximal muscle stimulation electrode was partially peeled back to allow room for the bar electrode. Single 1 ms pulses of electrical stimulation (DS7A, Digitimer Ltd., United Kingdom) were applied to determine the location and intensity of stimulation required to produce a twitch contraction at the tendon of the TA muscle without simultaneous activation of the antagonist muscles (Jochumsen et al., 2015b, 2016; Mrachacz-Kersting et al., 2016; Olsen et al., 2017). The electrode was secured with tape and the intensity determined was used in the subsequent ePAS intervention. Participants performed a short practice of ankle dorsiflexion movements with the visual cue which had been used in the MRCP recording. The blinded assessor then left the laboratory. Participants performed 50 repetitions of voluntary ankle dorsiflexion in time with the visual cue, while receiving single pulses of real or sham electrical stimulation to the dCPN. Each pulse of real electrical stimulation was timed to arrive in the M1 at the time of the peak negativity of the MRCP. The specific timing of electrical stimulation was achieved by calculating the participant’s peak negativity of the MRCP with respect to the visual cue, and subtracting 50 ms, to account for conduction time (Mrachacz-Kersting et al., 2007). The sham stimulation involved the same electrode set-up, but the stimulation output was switched off, so that the output light continued to flash but no stimulation was delivered. The interventions lasted approximately 15 min.

### Data Processing

#### TMS

TA MEP data was processed in Signal software (CED, United Kingdom), where the area of the rectified EMG signal was measured for each of the 10 MEPs in a 30 ms window from MEP onset, and then all 10 values were averaged.

#### MVIC Task

The peak amplitude of each MVIC (Klarner et al., 2014) was measured in Spike2 software (CED, United Kingdom). The force and TA EMG data collected during the MVIC task was then exported into LabVIEW 2017 software (National Instruments, United States) where the force data was low-pass filtered at 15 Hz (to eliminate high frequency noise artifact), and the EMG data was band-pass filtered (10–500 Hz). The time to 90% MVIC (Viitasalo and Komi, 1978; Canning et al., 1999) and the rate of force development (ROFD) 0–200 ms (Prieske et al., 2014; Maffiuletti et al., 2016) were calculated from the force data. ROFD 0–200 ms was calculated by dividing the force at 200 ms by the time (0.2 s). The root mean square (RMS) of the TA EMG signal was calculated 1-s either side of the peak force, and the peak amplitude (Ruiz Munoz et al., 2015) of the RMS signal was calculated for each of the three MVICs.

#### 30-s MVIC Task

For the 30-s MVIC task, the force data was exported into Signal software (CED, United Kingdom), and the maximum force and the area under the force–time curve from 5-s into the task to the end of the task (just prior to the final active muscle twitch) were measured (Schwid et al., 1999). Excluding the first 5-s eliminates the force generation phase (Schwid et al., 1999). If necessary, a slightly longer period was excluded to ensure the initial active twitch was not included. There was some variability in the duration of the fatigue task, and so to ensure the same level of fatigue was compared within each participant, the duration of data analyzed was reduced to the minimum available for each participant. This data was exported into Microsoft Excel where the AUC fatigue index was calculated as per equation A (Table 1) (Schwid et al., 1999). The AUC fatigue index is illustrated in Figure 4 by the blue area above the force–time curve, representing the force lost during the task. Also using Signal software (CED, United Kingdom), the resting and active muscle twitches in the force data were identified visually. The peak to peak amplitude of each twitch was measured. If there was any uncertainty about the onset of the twitch or its duration, the assessor considered the biologically feasible twitch duration and latency (Gandevia, 2001; Klass et al., 2005; Baudry et al., 2007), to ensure the twitch was measured, and not any pre- or post-twitch muscle activity. The twitch amplitudes were exported into Microsoft Excel software where VA, central fatigue, and peripheral fatigue were calculated as per equations E, G, and H (Gandevia et al., 1996).

Force and EMG data from the 30-s MVIC task were then exported into LabVIEW software (National Instruments, United States), where force data was low-pass filtered at 15 Hz, and EMG data was band-pass filtered (10–500 Hz). Both sets of data were divided into epochs, with the first epoch being a 400 ms-window before the initial active twitch, and then consecutive 1-s epochs between the initial and final active twitches. For both force and EMG data, the number of epochs was reduced to the minimum available for each participant. The mean force signal was determined in each epoch and exported into Microsoft Excel where the simple fatigue index was calculated as per equation B (Table 1) (Schwid et al., 1999; Sunnerhangen et al., 1999; Gerrits et al., 2009). The mean RMS values for each epoch were normalized to the peak RMS of the EMG recorded during the pre-intervention MVIC task. The percentage loss in EMG RMS during the fatigue task was calculated as per equation C (Table 1) (Mathur et al., 2005). In addition, each EMG epoch was further filtered with a Hanning window, and a Fast Fourier transformation using the LabVIEW Auto Power Spectrum VI (National Instruments, United States) computed the power spectrum of the EMG signal. The power spectrum was then integrated and the median frequency of each epoch was calculated. The percentage loss in median frequency of the EMG during the fatigue task was calculated as per equation D (Table 1) (Mathur et al., 2005).

### Data Analysis

The two primary outcomes, MVIC and the AUC fatigue index, were analyzed with univariate linear mixed models. The absolute values for the three repeated measures of MVIC were analyzed and the model estimated the post-intervention values for the three MVICs while controlling for baseline values. The fixed effects were ‘pre-intervention MVIC,’ ‘intervention’ (ePAS and sham), and ‘attempt’ (1, 2, and 3), and ‘participant’ was a random intercept effect. Individual MVIC data was also converted to percentage change values to allow comparisons with previous literature (Tanaka et al., 2011). The AUC fatigue index data was analyzed with a linear mixed model with fixed effects of ‘pre-intervention AUC fatigue index’ and ‘intervention,’ while ‘participant’ was a random intercept effect. By including the pre-intervention values for MVIC and the AUC fatigue index, the model estimated a fixed slope effect and adjusted for the different levels at baseline.

As the study was not powered for the secondary outcome measures, the primary and secondary outcomes were pooled in two multivariate linear mixed models, to improve statistical power (Schmitz et al., 1998). The first multivariate model included primary and secondary measures related to strength (MVIC, ROFD 0–200 ms, time to 90% MVIC, peak RMS during MVIC, VA). Whilst VA was collected during the 30-s fatigue task, it was collected at the start of the fatigue task which involves an MVIC, and therefore it was analyzed with the other MVIC measures. Outcomes which had three measures at each time point were averaged before being entered in the model. The second multivariate model included primary and secondary measures related to fatigue (AUC fatigue index, simple fatigue index, RMS EMG loss, median frequency loss, central fatigue, peripheral fatigue). For both multivariate models, the fixed effects were ‘intervention’ and ‘time’ (pre and post) for each outcome, and the random effects included a random intercept for ‘participant’ and a random intercept for ‘outcome measure within each participant.’ Both multivariate models estimated the effect of the interventions on the combined outcomes, as well as estimating the effect on each individual outcome. TMS-induced MEPs were only collected for one of the 13 participants who completed the protocol, and therefore were not analyzed at the condition level.

## Results

### Demographics and Protocol Completion

Participants were an average age of 69.9 years (SD 10.5 years) and an average of 6.4 years post-stroke (SD 4.8 years). Of the 15 participants, eight were male and 11 had a left hemiparesis. Participants used a variety of outdoor mobility aids (wheelchair *n* = 4, frame *n* = 2, quad stick *n* = 3, stick *n* = 2, unaided *n* = 4), indicating the range of lower limb disability in the sample. Optional TMS measurements were completed for two participants. Reasons for exclusion from TMS were history of seizures (*n* = 3), metalware (*n* = 3), migraines (*n* = 1), no resting MEP (*n* = 2), declined (*n* = 2), and unable to tolerate (*n* = 2). Of the fifteen participants, data for two participants was excluded due to failure to complete the protocol due to a communication impairment for one participant, and severe sleepiness for the other participant. In addition, methodological errors resulted in the loss of one participant’s EMG amplitude data for one intervention session, and another participants VA, central fatigue, and peripheral fatigue data for both intervention sessions.

### Primary Outcomes

The group data for MVIC (absolute and percentage change data) and the AUC fatigue index are presented in Table 2. Following the ePAS intervention there was a mean increase in ankle dorsiflexion MVIC of 7.33 Newtons (N) or an average percentage change of 9.75% from pre-intervention values. Following the sham intervention, absolute and relative changes in MVIC were negligible (+0.09 N and −0.17% change from pre-intervention). The univariate linear mixed model revealed a statistically significant difference between the ePAS and sham conditions (95% CI [0.08 N, 13.2 N], *t*[62.4] = 2.023, *p* = 0.047 for modeled data). The pre- to post-intervention change within the ePAS condition did not reach significance (95% CI [−3.2 N, 36.9 N], *t*[14.1] = 1.802, *p* = 0.093 for modeled data). For the AUC fatigue index, fatigue levels increased slightly after both the ePAS and sham interventions, with no significant difference between (95% CI [−6.6%, 5.4%], *t*[12.1] = −0.218, *p* = 0.831 for modeled data) or within conditions (ePAS 95% CI [−4.5%, 12.9%], *t*[19.3] = 1.010, *p* = 0.325, sham 95% CI [−3.0%, 12.6%], *t*[19.6] = 1.282, *p* = 0.215).

**TABLE 2:** Maximum voluntary isometric contraction (MVIC) and area under the curve (AUC) fatigue index data (primary outcomes).

**Outcome**	**ePAS**				**Sham**				**Between condition tests^+^**	
			
	**Pre mean (SD)**	**Post mean (SD)**	**Mean change (SD)**	**Within condition tests^+^**	**Pre mean (SD)**	**Post mean (SD)**	**Mean change (SD)**	**Within condition tests^+^**	**ePAS effect (95% CI)**	***p*-value**
**MVIC (N)**	137.73 (65.74)	145.06 (58.09)	+7.33 (18.24)	*p* = 0.093	146.38 (64.98)	146.48 (69.28)	0.09 (14.45)	*p* = 0.316	**+6.64 (0.08 to 13.2)**	0.047*
**MVIC (% change)**	100	109.75 (14.00)	+9.75 (14.00)	NA	100	99.83 (9.01)	−0.17 (9.01)	NA	NA	NA
**AUC fatigue index (%)**	23.17 (13.79)	24.03 (13.45)	0.86 (7.29)	*p* = 0.325	20.01 (7.44)	21.93 (11.31)	1.92 (7.64)	*p* = 0.215	−0.61 (−6.6 to 5.4)	0.831

**^+^**Derived from model estimates, *Statistical significance *p* < 0.05, NA, not applicable. The bolded data represents a statistically-significant result.

### Secondary Outcomes

The multivariate analysis of primary and secondary outcomes recorded during the MVIC task can be seen in Table 3. There was no statistically significant difference between the interventions on these combined outcomes (*p* = 0.14). However, ePAS had a significant effect on VA of 6.99 percentage units (*p* = 0.016). There were no significant effects on ROFD 0–200 ms, time to 90% MVIC, or peak EMG. The multivariate analysis of outcomes related to the 30-s MVIC task can be seen in Table 4 and showed no effect of ePAS on these combined outcomes (*p* = 0.53), and no significant effect on any individual outcomes.

**TABLE 3:** Multivariate analysis for outcomes related to maximum voluntary isometric contraction (MVIC) task.

**Outcomes**	**ePAS Mean (SD)**			**Sham Mean (SD)**			**Multivariate model**	
			
	**Pre**	**Post**	**Change**	**Pre**	**Post**	**Change**	**ePAS effect (95% CI)**	***p*-value**
MVIC (N)	137.73 (65.74)	145.06 (58.09)	+ 7.33(18.24)	146.38 (64.98)	146.48 (69.28)	0.09 (14.45)	+ 7.24(−2.88*to*17.36)	0.161
ROFD 0–200 ms (N/s)	257.81 (151.62)	224.93 (118.55)	−32.87(91.81)	254.43 (133.74)	236.78 (127.81)	−17.65(42.43)	−15.22(−58.74*to*28.30)	0.493
Time 90% MVIC (s)	1.68 (0.83)	1.69 (0.99)	0.01 (0.57)	1.43 (0.75)	1.60 (1.12)	0.17 (0.89)	−0.16(−0.54*to*0.23)	0.421
Peak RMS EMG during MVIC (V)	0.18 (0.15)	0.18 (0.10)	0.00 (0.04)	0.19 (0.13)	0.18 (0.13)	−0.01(0.02)	0.00(−0.03*to*0.04)	1
Voluntary activation (%)	81.87 (19.45)	85.36 (17.61)	3.49 (8.12)	87.37 (19.87)	83.86 (23.22)	−3.50(8.53)	**6.99 (1.30 to 12.68)**	0.016*

For multivariate linear mixed model of combined outcomes *p* = 0.14, *indicates statistically significant effect. The bolded data represents a statistically-significant result.

**TABLE 4:** Multivariate analysis for outcomes related to 30-s maximum voluntary isometric contraction (MVIC) task.

**Outcomes**	**ePAS Mean (SD)**			**Sham Mean (SD)**			**Multivariate model**	
			
	**Pre**	**Post**	**Change**	**Pre**	**Post**	**Change**	**ePAS effect (95% CI)**	***p*-value**
AUC fatigue index (%)	23.17 (13.79)	24.03 (13.45)	0.86 (7.29)	20.01 (7.44)	21.93 (11.31)	1.92 (7.64)	−1.06(−6.45*to*4.33)	0.700
Simple fatigue index (%)	11.27 (23.93)	21.72 (20.65)	10.45 (20.38)	18.73 (14.75)	20.28 (21.74)	1.55 (19.48)	8.90(−5.59*to*23.39)	0.229
Central fatigue (%)	9.72 (22.22)	17.26 (21.39)	7.54 (26.55)	17.83 (18.61)	9.59 (28.53)	−8.24(24.68)	15.77(−2.61*to*34.15)	0.093
Peripheral fatigue (%)	4.99 (13.32)	8.53 (15.62)	3.54 (12.74)	6.44 (7.27)	11.16 (9.59)	4.72 (7.82)	−1.18(−10.30*to*7.94)	0.800
RMS EMG loss (%)	25.00 (23.68)	31.74 (24.16)	6.74 (17.48)	22.44 (21.87)	35.51 (31.35)	13.07 (39.41)	−6.33(−26.43*to*13.77)	0.537
Median frequency loss (%)	7.42 (17.74)	4.19 (18.64)	−3.23(21.97)	2.77 (19.24)	4.43 (18.31)	1.67 (24.43)	−4.90(−20.72*to*10.93)	0.545

*For multivariate linear mixed model of combined outcomes *p* = 0.53.*

## Discussion

This study aimed to explore the influence of ePAS on a range of primary and secondary motor impairment and neurophysiological outcomes. Importantly, it is the first study to assess the immediate effects of ePAS with a sample of people with chronic stroke with a range of lower limb disability and using measures that do not require TMS.

The ePAS intervention resulted in a mean increase in absolute strength of 7 N, or an average percentage increase in strength of 10%. The univariate statistical analysis deemed this increase to be significantly different between ePAS and sham conditions (*p* = 0.047, Table 2). It was, however, noted that within the multivariate analysis, the between-condition comparison for MVIC was not statistically significant (Table 3). This is possibly due to the large within-participant variability estimated by the multivariate model; this possibility is supported by the larger standard error for MVIC in the multivariate model compared with the univariate model (MVIC standard error 5.163 and 3.347, respectively). Whilst multivariate analyses can result in greater statistical power for correlated measures (Schmitz et al., 1998), our results suggest that because a number of the secondary measures were not affected by the ePAS intervention, their inclusion in the multivariate model introduced additional measurement error which reduced statistical power for the MVIC measure. With regards to the clinical meaning of our strength findings, data for minimal clinically important differences in lower limb strength for people with stroke are not available; however, it has been suggested that individual change scores of 10–15% represent meaningful improvement (Lin et al., 2010). Thus, it is promising that within just a single session of ePAS, strength improvements reached the level deemed clinically meaningful. Our statistical analysis of muscle strength utilized absolute MVIC data, whereas previous neuromodulation studies in stroke have found significant effects on lower limb strength following tDCS using percentage change data (Tanaka et al., 2011; Sohn et al., 2013). From a clinical perspective, the use of percentage change data might be preferred because small absolute changes may have greater meaning to people with greater impairment. However, converting data to percentage change values is cautioned because it can distort the magnitude of change and violate the assumptions of the statistical analysis model (Vickers, 2001; Curran-Everett and Williams, 2015). Interestingly, when a univariate analysis was performed on our percentage change data, the between-condition comparison reached a higher level of statistical significance (*p* = 0.01). It was notable that of the 13 participants in the study, the three participants whose strength changes were less favorable following the ePAS intervention had the highest pre-intervention strength (>229 N). Thus, transforming the data resulted in smaller percentage change values for these three participants and provided more support for the ePAS intervention. This illustrates how the use of percentage change data can alter results and should be performed with caution (Curran-Everett, 2013; Curran-Everett and Williams, 2015).

There was large inter-individual variability in levels of pre-intervention impairment, with ankle dorsiflexion strength ranging from 57 to 262 N, or from minimal active movement to full range against resistance. The 10 participants whose MVIC changes were more favorable following the ePAS intervention were weaker (<187 N), whereas the three participants who responded less favorably had strength within the healthy range (Fraser et al., 2017). This might imply that people with stroke with greater impairment have more potential to benefit from ePAS. Other neuromodulation studies have reported greater improvements in those more impaired (Hummel et al., 2006; Stagg et al., 2012; Lefebvre et al., 2013; Oki et al., 2016). The method of pairing stimuli during ePAS may also be important in understanding variations in the intervention response. In previous ePAS research in healthy participants, voluntary ankle movements needed to be paired with muscle stimulation to have an excitatory effect, whereas pairing with nerve stimulation had no effect (Jochumsen et al., 2016). This may have occurred because voluntary movement produces higher levels of cortical activation (do Nascimento et al., 2006) which may require pairing with afferent volleys of higher frequency or amplitude, such as that produced with muscle stimulation. Thus, for the three least impaired participants in this study, a different ePAS intervention using muscle, rather than nerve stimulation, may have produced an increase in strength following the intervention. This notion of tailoring neuromodulation parameters to each individual’s level of impairment has been suggested in previous neuromodulation literature (Bradnam et al., 2012; Di Pino et al., 2014). Future ePAS research should have a sufficiently large enough sample size to allow stratification according to impairment level.

Another consideration is the method used to test strength. The TA maintains a low level of activity during quiet standing and produces short bursts of activity during the stance and swing phase of gait (Winter and Yack, 1987) and in challenging balance tasks (Tse et al., 2013). Therefore, the use of an MVIC may not be appropriate to assess improvements in the function of the TA muscle. In addition, given the task-specificity of neural plasticity (Kleim and Jones, 2008) an isometric contraction may not be sensitive to changes in corticomotor excitability associated with the ePAS intervention, which trains the ankle dorsiflexors in a phasic isokinetic contraction. Future studies should consider assessing strength using a phasic isokinetic muscle contraction, which more closely resembles the function of the TA and the movement trained during the ePAS intervention.

Although the between-condition univariate analysis revealed statistically significant effects on MVIC, the pre- to post-intervention changes within the novel-PAS condition did not reach significance (*p* = 0.093, Table 2). This may relate to an insufficient sample size. Data from previous tDCS work (Tanaka et al., 2011; Sohn et al., 2013) suggested that the sample size used in the present study would be sufficient to detect an immediate increase in muscle strength in people with stroke. However, other neuromodulation studies in people with stroke have failed to find statistically significant effects on strength (Hummel et al., 2006; Au-Yeung et al., 2014; Montenegro et al., 2016; Marquez et al., 2017). This contrast in the literature may be due to the use of different neuromodulation methods, the limb or muscle being targeted, or the large inter-individual variability in responses to neuromodulation (Sale et al., 2007; Muller-Dahlhaus et al., 2008; Ridding and Ziemann, 2010; Lopez-Alonso et al., 2014). Further research is required to determine if the immediate strength improvements following ePAS seen in this study can be confirmed in a larger sample of people with stroke.

The findings revealed a significant effect of ePAS on VA (7 percentage units). This effect was seen with only a single session of ePAS. In contrast, other neuromodulatory research has failed to show significant effects on VA following a single session (Urbach et al., 2005; Kumpulainen et al., 2015; Angius et al., 2016a, b), but a 4-day neuromodulatory intervention has increased VA (Frazer et al., 2016). The VA findings confirm that ePAS has a central mechanism, and fit with previous findings of its effects on corticomotor excitability in healthy people (Mrachacz-Kersting et al., 2012; Niazi et al., 2012; Jochumsen et al., 2015b; Olsen et al., 2017) and people with stroke (Mrachacz-Kersting et al., 2016, 2019). It is assumed that the mechanism for increased VA is supraspinal, as previous ePAS work has shown no effect on spinal cord excitability (Mrachacz-Kersting et al., 2012; Niazi et al., 2012). In terms of the magnitude of change, the 7 unit increase in VA percentage exceeds the error of the measurement (1.3–3.8%) reported in lower limb muscles (Todd et al., 2004; Signal, 2007). It is not clear whether this change in VA has functional consequences; however, the link between VA deficits after stroke and poor functional performance (Horstman et al., 2008) suggests this is possible.

Muscle power, investigated as a secondary measure using the ROFD 0–200 ms and time to 90% MVIC, was not significantly increased by a single session of ePAS. While previous tDCS research has shown improvements in muscle power in healthy people (Lattari et al., 2017), immediate improvements in muscle power may be more difficult to achieve in people with stroke, due to non-neural factors such as antagonist plantarflexor muscle stiffness (Maffiuletti et al., 2016). Changes in muscle power may require more than a single session of ePAS, as well as other standard interventions to address non-neural changes (Gao et al., 2011).

The results for total neuromuscular fatigue (measured with the AUC fatigue index and the simple fatigue index) and central fatigue were not statistically significant, suggesting that a single session of ePAS has no effect on fatigue. It has been proposed that neuromodulatory interventions might prevent central fatigue by reducing the intracortical inhibition which acts on the M1 during sustained exercise (Taylor and Gandevia, 2001; Cogiamanian et al., 2007; Vitor-Costa et al., 2015). However, there was no evidence of this preventative effect in the present study. The effects of neuromodulatory interventions on neuromuscular fatigue are inconsistent (Milanovic et al., 2011; Kumpulainen et al., 2015; Angius et al., 2017) and studies which have specifically investigated central fatigue, have not shown significant effects in healthy people (Kumpulainen et al., 2015; Angius et al., 2016b). As for the MVIC testing method, an alternative method for testing neuromuscular fatigue may have been more suited to the function of the TA. While phasic and low-load isometric fatigue protocols are more time-consuming they should be considered for future research into the effects of ePAS on neuromuscular fatigue.

There was no significant effect of ePAS on EMG amplitude during the MVIC. This contrasts with previous work which has shown that peak EMG amplitude during an MVIC can significantly increase following tDCS, in line with increases in MVIC (Krishnan et al., 2014). There were also no significant effects of ePAS on EMG amplitude and median frequency during the 30-s MVIC, although for both interventions, these measures declined, which is in line with fatigue-related changes in motor unit activity in healthy adults (Bigland-Ritchie and Woods, 1984; Rubinstein and Kamen, 2005; Babault et al., 2006). These findings may be related to the insensitivity of EMG measures to detect small changes associated with neuromodulation, as demonstrated by tDCS studies which showed improved neuromuscular fatigue without any effects on EMG amplitude during the fatigue task (Cogiamanian et al., 2007; Abdelmoula et al., 2016). Further research is needed to determine if ePAS and other neuromodulatory interventions have an effect on EMG measures.

As an exploratory study, a large number of outcome measures were investigated, and these were dealt with by performing two multivariate analyses. The VA measurement technique also has some limitations, as the interpolated twitch technique does not represent true levels of VA and is less sensitive at higher levels of MVIC (Belanger and McComas, 1981; de Haan et al., 2009); this may have resulted in an underestimation of VA changes for those with higher levels of strength. Another limitation was that VA was measured at the start of the fatigue task, and although participants were advised to contract maximally, they may have used submaximal effort in anticipation of the task ahead, which might have reduced their VA. This could be avoided in future by recording VA during a brief MVIC. A further limitation of this study was that participants were recruited though the community, which meant the sample could not be described with respect to lesion size or location. The sample was heterogenous in terms of chronicity and severity of lower limb disability, which improves external validity, but limits our understanding about the efficacy of ePAS in certain types of stroke.

## Conclusion

In conclusion, this study demonstrated that a single session of ePAS can significantly increase isometric muscle strength and VA in people with chronic stroke, more than an attention- and dose-matched sham intervention. The findings confirm the neuromodulatory effect of ePAS and provide support for the future investigation of ePAS as an adjunct to standard stroke rehabilitation, applied either before or during other interventions. Improving the central nervous system drive to a muscle with ePAS has potential to improve motor performance during standard rehabilitation tasks, such as task-specific training or strength training, and with repetition has potential to lead to improved motor recovery (Kleim and Jones, 2008). Future research should investigate the effects of applying ePAS in combination with standard rehabilitation techniques.

This study was the first to explore the effects of ePAS on a range of outcome measures other than corticomotor excitability, and also to recruit a sample with a range of lower limb disability. This study demonstrates that the effects of ePAS can be investigated with measures other than TMS and provides guidance for the selection of outcome measures in future research. In the present study, changes in muscle power and neuromuscular fatigue were not significant. However, significant changes in these measures may require more than a single session of ePAS, and therefore future research should investigate the effects of multiple sessions of ePAS.

## Data Availability Statement

The datasets for this manuscript are not publicly available because the ethics approval did not include permission to share data. Requests to access the datasets should be directed to SO (Sharon.Olsen@aut.ac.nz) who can request ethical approval to access the data.

## Ethics Statement

This study was reviewed and approved by the Health and Disability Ethics Committees (17/NTB/80). All participants provided their written informed consent to participate in this study.

## Author Contributions

SO, NS, and DT conceived the study design with input from IN, GA, and MJ. SO managed the project and recruited all participants. SO, RN, and IN managed data collection. SO and GM managed data processing, and UR contributed to the statistical analysis. All authors contributed to data interpretation. SO prepared the manuscript draft with intellectual input from all other authors. All authors approved the final manuscript.

## Conflict of Interest

The authors declare that the research was conducted in the absence of any commercial or financial relationships that could be construed as a potential conflict of interest.

## Footnotes

1 seniam.org
